# Supplementary material for: Acute effects of commercial group exercise classes on arterial stiffness and cardiovagal modulation in healthy young and middle-aged adults: A crossover randomized trial
Source: PLoS One. 2025 Mar 13;20(3):e0319130. doi: 10.1371/journal.pone.0319130 (PMC11906072; doi:10.1371/journal.pone.0319130)
Supplement: S1 Supplement — Data depicted as mean (SD). Abbreviations: PP, Pump Power; GT; Global Training; REE, resting energy expenditure; EE, energy expenditure; TEE; total energy expenditure; HR, heart rate; HRR, heart rate reserve; VO2 RR, oxygen uptake reserve; MET, metabolic equivalent. #Different from Young Adults in the corresponding fitness class (p < 0.05); * Different from BIKE within groups (p < 0.05). †Different from PP within groups (p < 0.001). (PDF) [file pone.0319130.s001.pdf]

With this document we would like to ask the Research Ethics Committee of Faculdade de Motricidade Humana – Universidade de Lisboa for an ethical review of the research entitled "PUMPING ARTERIES – Acute Effects of Exercise Mode on Arterial Stiffness and Cardiac Autonomic Function in Healthy Young Adults". In this document you will find relevant topics for the ethical appraisal of PUMPING ARTERIES.

Application for Ethical Appraisal

PUMPING ARTERIES – Acute Effects of Exercise Mode on Arterial Stiffness and Cardiac Autonomic Function in Healthy Young Adults

Xavier Melo & Helena Santa-Clara

DESCRIPTIVE ELEMENTS OF THE APPLICATION 3
TITLE 3
NAME OF THE PROPONENT 3
PRINCIPAL RESEARCHERS 3
CONTACT 3
RECRUITMENT START DATE 3
RECRUITMENT DUE DATE 3
STUDY DUE DATE 3

DESCRIPTIVE ELEMENTS OF THE PROJECT 4

PURPOSE 5

METHODOLOGICAL PROCEDURES 5
PARTICIPANTS 5
DESIGN 5
INTERVENTION SESSIONS 7
HEALTH-RELATED PHYSICAL FITNESS 7
ENERGY EXPENDITURE 8
ADVANCED HEMODYNAMICS 9
ARTERIAL STIFFNESS 10
AORTIC AND LIMB ARTERIAL STIFFNESS 10
CAROTID BLOOD PRESSURE 10
CAROTID ARTERIAL STIFFNESS INDICES 10
CAROTID BLOOD PRESSURE WAVE INTENSITY 11

BENEFITS 12
IMPACT OF PROJECT ON PARTICIPANTS AND PARTICIPATING ORGANIZATIONS 12

PRIVACY AND CONFIDENTIALITY 13

STORAGE 13

STATISTICAL ANALYSIS AND DATA MONITORIZATION 13

COMPENSATION AND INSURANCE 14

END-OF-STUDY PROCEDURES 14

CONFLICT OF INTEREST 14

REFERENCES 15

Application for Ethical Appraisal

PUMPING ARTERIES

Descriptive Elements of the Application

Title

- Acute effects of Exercise Mode on Arterial Stiffness and Cardiac Autonomic Function in Healthy Young Adults
- Efeitos Agudos do Modo de Exercício na Rigidez Arterial e Função Autonómica Cardíaca em Adultos Jovens Saudáveis

Name of the Proponent

- Xavier Melo | PhD | Ginásio Clube Português

Principal Researchers

- Xavier Melo | PhD | Ginásio Clube Português
- Helena Santa-Clara | PhD | Faculty of Human Kinetics – University of Lisbon.

Contact

Name: Xavier Melo | PhD | Ginásio Clube Português

Email:

Telephone:

Recruitment Start Date

January 2020

Recruitment Due Date

November 2021

Study Due Date

June 2022

Descriptive Elements of the Project

Large artery distensibility is physiologically important for cardiovascular efficiency. Distensible large arteries reduce impedance to systolic ejection and cardiac work, slows pulse wave velocity (PWV) so that the return of reflected pressure waves is delayed until after aortic valve closure, and favors coronary perfusion during diastole [1]. Conversely, stiffening of the central arteries results in an elevation in systolic blood pressure (SBP) and a lowering of diastolic blood pressure (DBP) which, in turn, increases left ventricular afterload and alters coronary artery perfusion [2]. These changes may result in left ventricular hypertrophy [3] and increased fatigue of arterial wall tissues [4], all of which substantially increase the risk of cardiovascular events and all-cause mortality [5, 6].

The sympathetic nervous system is a key regulator of blood pressure, and abnormal activity in this system is related to cardiovascular risk [7]. Specifically, indices of cardiac autonomic function such as heart rate recovery (HRR) and heart rate variability (HRV) are associated with brachial and aortic blood pressure [8, 9]. When measured after a maximal exercise effort, HRR and HRV are considered powerful independent predictors of mortality in healthy subjects and in various clinical populations [10-13].

In addition to their ability to predict cardiovascular event risk, arterial stiffness and indices of cardiac autonomic function are predictive of the success of different therapies [14, 15]. Cardiovascular medications including beta-blockers have been reported to reduce aortic PWV and wave reflection [14], most likely due to reductions in sympathetic activity [9, 16]. Additionally, a single bout of exercise has been shown to reduce both arterial stiffness (i.e., aortic PWV [17, 18]) and wave reflection (i.e., AIx and reflection magnitude; [19, 20]), and significantly impact on indices of cardiac autonomic function [18, 21-23].

This emerging area of research concerning “the physiology of recovery” may provide insights that could help optimize exercise recommendations for health and performance [24]. For example, not only some adaptations to physical training appear to result from the summation of acute effects of exercise bouts [25], as exercise-induced changes may be influenced by exercise mode with several studies reporting variable results [18, 26, 27]. HRR and HRV after maximal CPET vary according to whether exercise is performed on treadmill or cycle ergometer [28-30], since the physiological strain induced by treadmill exercise seems to be significantly greater than in cycle ergometer [31-33]. As for arterial stiffness, only a limited number of studies have directly compared exercise-induced acute changes in arterial stiffness (i.e., aortic PWV) following different exercise modes with discrete responses following each mode identified [18, 26].

Overall, these studies have focused on exercises rather on activities, to represent typical sessions in accordance with current guidelines for improvement and maintenance of cardiovascular health [34]. An obvious disadvantage rests on their reduced ecological validity [35, 36]. Participation in group fitness classes is increasingly common method by which individuals may fulfil the criteria set down in the ACSM guidelines for enhancing and maintaining cardiovascular fitness [34]. These can appropriately function to enhance measures of cardiorespiratory fitness when performed for at least 3 days/week for 30 minutes and induce favorable changes in cardiovascular risk profile [1-6]. The popularly of group fitness classes with the general public has been demonstrated for more than two decades and is most likely due to the social and non-competitive class environment. A broad range of group fitness classes are now available in both commercial and community fitness centers that incorporate a wide range of exercise modalities (e.g. stationary cycling, step classes, group resistance exercise, pilates and aerobic dance) using a combination of music and instructor choreographed routines and designed to accommodate varying fitness levels. However, despite the high participation rates of group fitness classes, no available data exists that describes and/or compares the vascular and autonomic responses and/or metabolic costs of different group fitness classes. In addition,

the extent to which participation in such classes may assist in the improvement of vascular and autonomic function, and whether there are periods of significant concern eventually caused by transient changes in arterial stiffness or autonomic function immediately following exercise.

Purpose

The aim of this study was to compare the time-course of post-exercise parasympathetic reactivation of the heart and changes in local and regional indices of arterial stiffness to different fitness classes (Bike, Pump Power and Global Training), as prescribed for health [34] and commercially available.

Methodological Procedures

Participants

Twelve apparently healthy adults aged between 21 to 34 years will be included in this study. These will be recruited from the professional network of GCP. We do not expect these adults to be enrolled in any other scientific study besides PUMPING ARTERIES. Informed written consent will be signed by volunteers prior to the first evaluation day (Figure 1) in accordance with approval by the Ethical Committee of Faculdade de Motricidade Humana – Universidade de Lisboa. We expect recruited participants to be outwardly active with some experience in both aerobic and resistance exercise (~3–4 times/week, >3 months). No trained athletes will be included in the cohort. All participants will be healthy or perceived to be healthy based on the sport's medical examination or the preparticipation screening process. Exclusion criteria include any form of cardiovascular disease, more than one cardiovascular disease risk factor, resting hypertension (systolic blood pressure >140 mmHg, diastolic blood pressure > 90 mmHg), any prescription medication use, and currently smoking.

Design

The study is constructed as a randomized, cross-over, repeated-measures intervention. Participants will attend 4, separate, intervention sessions consisting of one group class of Bike, Pump Power, Global Training or no exercise (CON). There will be a minimum of 72 h between sessions [18, 37], and each session will consist of initial rest, experimental intervention, and recovery (Figure 1). Participants will be advised not to engage in any strenuous physical activity at least 48 hours before the study days. Body composition and cardiorespiratory fitness for each participant will be evaluated prior to- and following to- the CON session, respectively.

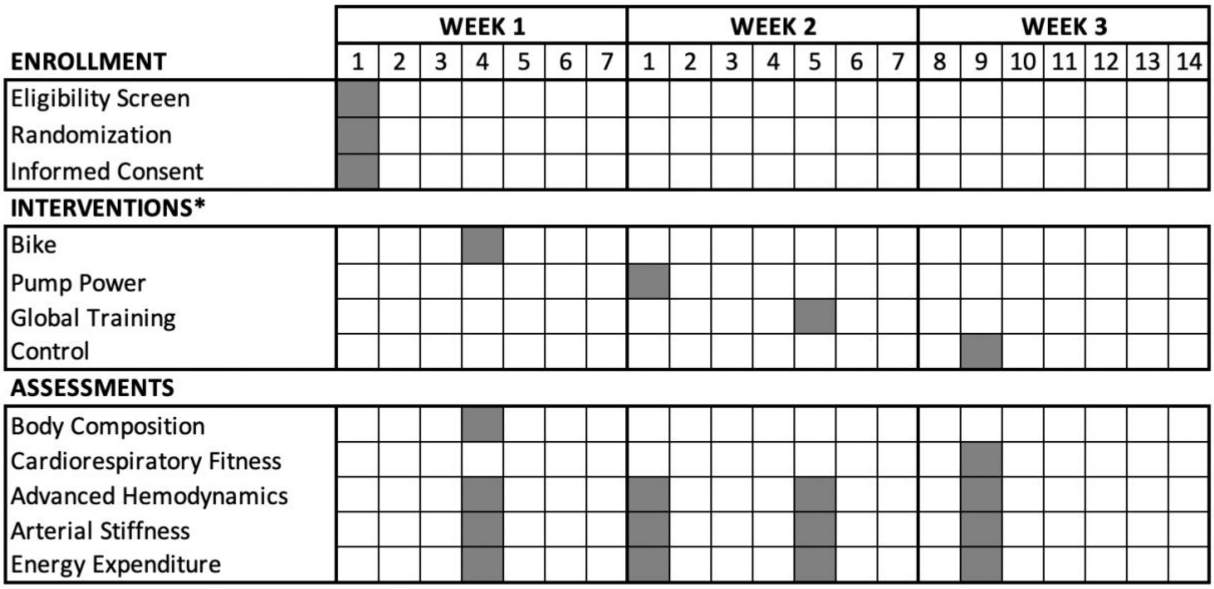

FIGURE 1: TIMELINE AND MAIN OUTCOME VARIABLES OF PUMPING ARTERIES

** INTERVENTIONS WILL BE RANDOMIZED. THE TIMELINE HEREIN DISPLAYED IS MERELY A GRAPHIC REPRESENTATION OF A PARTICIPANT'S ACTIVITIES FOLLOWING RANDOMIZATION.*

In each session, participants will initially undertake 20-min of supine rest on a cushioned examination table with resting energy expenditure measured by indirect calorimetry (K5, Cosmed, Rome, Italy) and heart rate and blood pressure recorded continuously using digital plethysmography technique (Finapres® Nova, Ohmeda, 2 Louisville, Colorado, USA), followed by regional assessments of PWV and pulse wave analysis of the aortic, brachial and femoral arteries on the right side of the body using applanation tonometry (Complior Analyze, ALAM Medical, Paris, France), and carotid arterial stiffness indices and wave intensity analysis using an ultrasound (Arietta V60, Hitachi Aloka Medical Ltd, Mitaka-shi, Tokyo, Japan). In the time following, participants will perform one of three exercise sessions (Bike, Pump Power or Global Training). Exercise sessions will consist of either a 45-minute class of Bike, Pump Power, and Global Training, while energy expenditure will be continuously measured by indirect calorimetry (K5, Cosmed, Rome, Italy) (Figure 2). These sessions are characterized by distinct metabolic demands, representing the typical sessions provided by gyms and health clubs to improve or maintain cardiovascular health. In the control condition, the participants will remain quietly seated for 45-min, maintaining a good posture. Only one participant will be tested per class with approximately 10-20 non-research participants also performing each group fitness class alongside the research participants. Upon completion of each intervention/session, participants will return immediately to the examination table and recover in the supine position for 30-min while hemodynamic and stiffness indices are reevaluated at 10, 20- and 30-min following recovery and compared to those at rest.

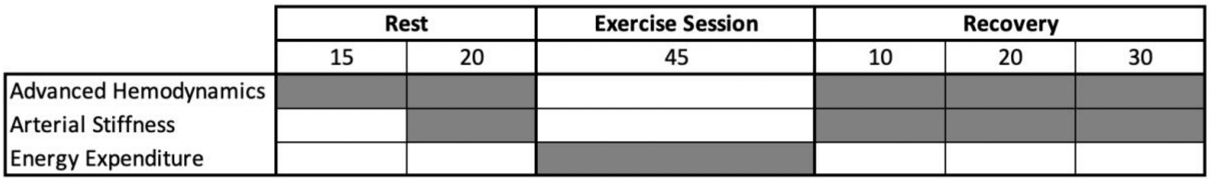

FIGURE 2: EXPERIMENTAL DESIGN OF PUMPING ARTERIES

Participants will be blinded to the order of the experimental interventions until arrival at the laboratory. All sessions will be conducted in the morning with each participant performing sessions at the same time of the day to minimize any potential diurnal variation. Participants will be instructed not to ingest any food or drink (except water) 4h before the sessions, and to avoid alcohol, caffeine, and vigorous exercise for at least 12h preceding each session [38].

Intervention sessions

A lack of consistency with choreography, and other variables, such as the type of step, the level of impact of the step, arm movements and cadence of music, and intensity of a class will impact on the ability to use the results from previous research. Research involving pre-choreographed programs can control some of bias derived from the cadence of music, and intensity of a class by establishing identical music and choreography with instructors following strict guidelines and delivering the same choreography to the same music, thus standardizing class and exercise performance. While there is no specific mention of group classes in the American College of Sports Medicine (ACSM) Position Statement concerning Cardiorespiratory Fitness in apparently healthy adults [39], these appear to fulfil the criteria set down in the ACSM guidelines for enhancing and maintaining cardiovascular fitness.

The Bike session consists of a rhythmic indoor cycling class, with fluctuations in intensity set to changes in position, music rhythm, cadence, and revolutions per minute. It is an activity characterized by steps of workout with variable intensity and a high/moderate involvement of the cardiovascular system as well as the skeletal muscles [40]. Participants will be advised to strictly adhere to the verbal cues from the instructor when told to adjust cycling cadence and resistance. Pump Power is intended to provide a comprehensive total body weight-training program aiming to improve strength, muscular endurance and general fitness. The class is choreographed to music and uses a combination of barbells, body-weight exercises and free-weight plates. Participants select weights based on the target muscle group for the specific song or track and their own personal goals. The Global Training class encompasses both aerobic and resistance components, combining athletic movements like running, lunging and jumping with strength exercises such as barbells, body-weight exercises and free-weight plates. All classes will be 45 minutes long and contain 10-12 'tracks' or songs, each lasting from 4–7 minutes. The choreography of the music plays an important role because it may modify the participant's motivation and the intensity of the exercise. The classes start with an opening warm-up, followed by eight tracks, each targeting specific muscle groups, finishing with a static stretch cool down.

Health-related Physical Fitness

Body Composition

Height and sitting height will be measured to the nearest 0.1 cm and body weight will be measured to the nearest 0.1 kg on a scale with an attached stadiometer (model 770, Seca; Hamburg, Deutschland). Body mass index (BMI) will be calculated as body mass divided by height squared ($kg.m^{-2}$). Waist circumference

(WC), an estimate of the subcutaneous and intra-abdominal (i.e. visceral) adipose tissue in the abdominal region [41] will be measured to the nearest millimeter with an inelastic flexible metallic tape (Lufkin - W606PM, Vancouver, Canada) midway between the lower rib margin and the iliac crest.

Body composition will also be measured with a seca mBCA 515 using four pairs of electrodes (eight electrodes in total) that are positioned at each hand and foot. The 8-electrode technique enables segmental impedance measurement of the arms and legs. Impedance is measured with a current of 100 µA at frequencies between 1 and 1 000 kHz. The mBCA 515 is designed for measurements in the standing position and consists of a platform with an integrated scale, a handrail system and a display and operation unit. Each side of the ascending handrail carries six electrodes of which two were chosen depending on person's height. According to the manufacturer´s instructions, arms should be held straight.

All measurements will be performed at GCP by the same researcher at each measurement round.

Cardiorespiratory Fitness

Each participant will perform a ramp incremental cycle ergometer test to exhaustion on a calibrated electronically braked cycle ergometer (Monark 839 E, Ergomedic; Monark, Vansbro, Sweden) at a pedal cadence of 70 to 75 rev.min^{-1} using an electronically braked cycle ergometer. Initial and incremental workloads will be 30-40 W.The seat will be adjusted so that the participant's legs could be at near full extension during each pedal revolution.

Inspired and expired gases will be continuously analyzed, breath-by-breath, through a portable gas analyzer (K5, Cosmed, Rome, Italy). Before each test, the O_2 and CO_2 analyzers are calibrated using ambient air and standard calibration gases of known concentration (16.7% O_2 and 5.7% CO_2). The calibration of the turbine flowmeter of the K5 is performed using a 3-l syringe (Quinton Instruments, Seattle, Wash., USA) according to the manufacturer's instructions. Heart rate will be continuously monitored (Garmin, US). The participants will not carry the gas analyzer. This compact device is easy to attach without constricting the participants movements. Data will be evaluated in 10-second averages, and peak VO_2 will be defined as the highest 10-second value attained in the last minute of effort provided 2 of the following criteria are met: (1) Attaining ~90% of age-predicted maximal heart rate; (2) Plateau in VO2 with an increase in workload (<2.0 mL.kg-1.min-1); (3) Rating of perceived exertion ≥ 18 (6-20) and; (4) Respiratory exchange ratio ≥ 1.1; (5) subjective judgment by the observer that the participant could no longer continue, even after encouragement.

All measurements will be performed at GCP by the same researcher at each measurement round.

Energy Expenditure

The nature of group fitness classes presents several challenges for research aimed at measuring exercise-induced energy expenditure and hence the choice of both a valid and minimally invasive measuring device is critical for useable results. Therefore, expired gases at rest and during exercise will be continuously analyzed following thoroughly mixing prior to being sampled, through an open-circuit indirect calorimetry portable gas analyzer (K5, Cosmed, Rome, Italy) which has been previously validated [42]. Before each test, the O_2 and CO_2 analyzers will be calibrated using ambient air and standard calibration gases of known concentration (16% O_2 and 5% CO_2). Both the calibration of the flowmeter using a 3-l syringe (Quinton Instruments, Seattle, Wash., USA) and the scrubber calibration will be performed according to the manufacturer's instructions. Heart rate will be continuously monitored (Garmin, US). This compact device is easy to attach without constricting the participants movements. Data will be analyzed in 1-min averages.

Advanced Hemodynamics

Individuals will be monitored non-invasively with a Finapres® Nova (Ohmeda, Louisville, Colorado, USA) (volume-clamp technique) in all measurement rounds. In supine humans, the finger arterial pressure signal can be modelled to a reconstructed pressure curve, using a general inverse anti-resonance model [43]. Subsequently, the device calculate left ventricular stroke volume (SV) and use this with HR to provide a calculated measure of CO. Based on reconstructed Q waveforms, the Windkessel model parameters (R, C and ZC) will then be assessed by the system [44]. Plethysmography sensors will be placed in the second and third finger of the right hand. An oscillometric cuff will then be attached to the same arm and the device calibration is completed. Heart rate signal (HR) will be computed from a I lead digitized electro-cardiogram (ECG), included in the equipment. Individuals will be studied for 15 minutes.

Effective Arterial Elastance

Effective arterial elastance is defined as a steady-state arterial parameter that incorporates the principal elements of vascular load. It can be calculated according published equations expression [45]:

$$Ea = \frac{R + Z_C}{T_S + RC\left(1 - e^{-\frac{T_D}{RC}}\right)}$$

where C is a compliance element that mimics the elastic and buffering properties of the large arteries, R is a resistive element that represents de resistance generated by the small arteries and arterioles, Zc is a second resistive element that represents the impedance of proximal aorta [46], and TS and TD are the systolic and diastolic periods, respectively. TS will be obtained as the time between the point before the abrupt rise of arterial pressure waveform and the dicrotic notch. Finally, ventricular end-systolic pressure (ESP) was approximated as:

$$ESP = SV.Ea$$

Heart rate variability

R-R intervals are derived from beat-to-beat blood pressure pulse intervals using finger plethysmography (Finapres® Nova, Ohmeda, Louisville, Colorado, USA) in all measurement rounds. Finger plethysmography-derived peak-to-peak intervals are highly correlated with ECG R-R intervals, with similar variability. The up-stroke is determined using the pressure signal with a resolution of 2 ms, and the interval between the two consecutive upstrokes is measured. In the frequency domain, the two primary components are low-frequency (LF; 0.04–0.15 Hz) and high-frequency (HF; 0.15–0.40 Hz) spectra. Heart Rate Variability measures provide information primarily on vagal modulation with the LF power spectrum reflecting both sympathetic and parasympathetic modulation and HF acting as a surrogate marker of parasympathetic modulation of the R-R intervals [47]. The LF/HF ratio is used as an indicator of sympathovagal dominance [48]. All data acquisition and post-acquisition analyses will be carried out in accordance with the Task Force of the European Society of Cardiology and North American Society of Pacing and Electrophysiology [49].

Baroreflex Sensitivity

We will use spectral method to compute baroreflex sensitivity as the transfer gain of the cross-spectra between pressure and interval in all measurement rounds. Their coherence is usually high in the10 s rhythm band taken from 0.06 to 0.15 Hz and at ventilatory frequencies in the spectra between 0.15 and 0.5

Hz. Spectral estimates of the entire recording will be computed with the device software (Finapres® Nova, Ohmeda, Louisville, Colorado, USA), providing an easy-to-use interface [50]. A discrete Fourier transform will be used that needs no interpolation or zero padding. Triangular spectral smoothing will be set at a width of 10 for this study, in view of the 10 min duration of the records. Spectral density, coherence, pressure–interval transfer gain and phase plots are shown on the device screen and in addition a cursor allows manual selection of bands in which coherence and spectral power are high. An output program lists the resultant data and all the choices made for later analysis.

Brachial Blood Pressure

The brachial systolic blood pressure (SBP) and diastolic blood pressure (DBP) will be measured with the participants in the supine position using an automated oscillometric cuff (Bosotron 2®, Bosch & Sohn, Germany) in all measurement rounds. Two measurements will be taken and if these values deviated by >5 mmHg, a third measurement is performed. The average of the two closest values will be used.

Arterial Stiffness

Aortic and Limb Arterial Stiffness

Arterial stiffness as measured by pulse wave velocity (PWV) obtained by applanation tonometry will be measured in all measurement rounds. A single operator locates the carotid, femoral, radial and distal posterial tibial arteries on the right side of the body and mark the point for capturing the corresponding pressure curves with two specific pressure sensitive transducers. The distance between the carotid and femoral, radial and distal posterial tibial arteries is measured directly and entered into the Complior Analyse software (ALAM Medical, Paris, France). Right brachial blood pressure is measured and entered into the Complior Analyse software, and then signal acquisition is launched. The operator positions the carotid sensor with the help of its specific holder and manually held the femoral sensor on the femoral artery and the distal sensor in the distal posterial tibial artery. When the operator observes 10 carotid pulse waveforms of sufficient quality, simultaneous carotid and femoral, radial and distal posterial tibial pressure curves are recorded for 10 pulse waveforms. The time delay (aortic transit time) between the two pulse waveforms is then calculated automatically. Values obtained from the carotid to femoral artery, carotid to radial artery and carotid to distal posterial tibial artery will be taken as indices of central/aortic, upper and lower limb arterial stiffness, respectively.

Carotid Blood Pressure

Carotid Systolic Blood Pressure (cSBP) will also be assessed by applanation tonometry (Complior Analyse) in all measurement rounds, from right carotid traces acquired during the carotid pulse wave velocity (cPWV) assessment. The waveforms will be averaged and the mean values will be extracted from 15 s window of acquisition. The carotid waveforms are calibrated from MAP and bSBP, measured immediately before the acquisition.

Carotid Arterial Stiffness Indices

The severity of Cardiovascular disease is likely to be reduced if atherosclerosis is detected early and before morphologic changes such as plaque and wall thickening are visible in the arterial walls. Prevention and treatment of lifestyle-related diseases are increasingly important today, and so the role of the ultrasound diagnostic system is not only to observe the morphologic changes but also to perform functional assessment. Hitachi Aloka realized functional assessment of arteries by developing our unique eTRACKING technology.

eTRACKING achieves highly precise measurements of the vessel distension in real time, using radio frequency (RF) signals. Conventionally, vessel diameters are measured visually on B-mode and M-mode images, this can lead to variations in the measurement because it is difficult to know time-dependent changes in the vessel diameter on B-Mode. Furthermore, it is difficult to identify exact timing for the measurement of the maximum and minimum diameter. To overcome these problems in measuring vessel diameter, eTRACKING has been developed and implemented in a diagnostic Ultrasound system.

Common carotid diameter values and diameter-derived pressure data will be used to calculate established indices of local arterial stiffness, such as the Peterson's pressure-strain elastic modulus (ε) and the stiffness index (β), according to published algorithms [51], in all measurement rounds.

$$\varepsilon=[(Ps-Pd)/(Ds-Dd)]\times Dd,$$

and

$$\beta=\ln(Ps/Pd)/(Ds-Dd)/Dd,$$

where Ps and Pd are systolic and diastolic pressure, respectively, and Ds and Dd are the maximum and minimum arterial diameters measured by wall tracking of the intima-media borders of the carotid artery.

The augmentation index (AIx), a surrogate measure of arterial wave reflection from the periphery, expressed as a ratio of the “augmented pressure” (*i.e.*, the pressure difference between the “shoulder” of the carotid artery pressure curve and the peak carotid systolic pressure) to the pulse pressure, will also be calculated from the diameter-derived pressure waveform using the third derivative method to identify the carotid pulse inflection point [52].

Carotid Blood Pressure Wave Intensity

The heart and the arterial system constantly interact with each other through forward travelling waves and reflected waves. Wave Intensity (WI) is calculated as the product of the derivatives of the simultaneously recorded blood-pressure changes and blood-flow-velocity changes. Wave Intensity can be obtained at an arbitrary point in the circulatory system. Blood pressure change waveform is conventionally measured invasively. Hitachi has developed a system to calculate blood pressure change noninvasively and with a high level of accuracy. Based on the similarity of the cyclic blood pressure change and its simultaneous vessel diameter change, a blood pressure waveform is derived non-invasively. This non-invasive pressure waveform can then be analyzed further. WI is a new hemodynamic index which is potentially useful for analyzing interaction of the cardiovascular system, including contraction and dilatation characteristics, influence of reflected waves from peripherals, interaction of elastic and resistance vessels and an index related to time.

The right common carotid artery will be scanned with a Arietta V60 ultrasound machine (Hitachi Aloka Medical Ltd, Mitaka-shi, Tokyo, Japan) using a 7.5-MHz linear array probe incorporating a 5-MHz Doppler transducer in all measurement rounds. In longitudinal view, the probe will be manipulated so that the intima of the artery is imaged clearly from both the anterior and posterior walls, and a single scan line is aligned perpendicularly to the vessel walls at a site 20 mm proximal to the carotid bulb. On-screen cursors will then be placed on the anterior and posterior intima-media borders to enable tracking of both walls. The corresponding displacement waveforms and diameter curve will thus be calculated using high-resolution online wall tracking (“E-track” technology), with a sampling rate of 1 kHz.

Arterial pressure waveforms will be obtained automatically in real time by calibrating peak and bottom values with systolic and diastolic blood pressure measured with sphygmomanometry, as previously validated [53].

Next, a pulse wave Doppler ultrasound beam will be aligned to the vessel walls at the site of acquisition of the diameter waveform, to simultaneously acquire velocity data from a color flow Doppler box covering the entire vessel lumen at an angle of insonation between 60 and 65 degrees, with a sampling rate of at least 50 Hz. Spatially-averaged flow velocity will be calculated from the mean of the color Doppler data [54]. Arterial diameter and velocity will be recorded continuously for 20 s. After completing the acquisition, all data is displayed and any individual beats with noisy or unrepresentative waveforms will be rejected; all other beats (typically about 20) will be selected and signal-averaged to give single waveforms of diameter and velocity [55]. Averaged vectors for pressure and velocity, together with the electrocardiogram, and calculated net WI will be automatically displayed by the software.

Benefits

Impact of project on participants and participating organizations

Impact of PUMPING ARTERIES on the participants

The health-related components of physical fitness have a strong relationship with overall health, are characterized by an ability to perform activities of daily living with vigor, and are associated with a lower prevalence of chronic disease and health conditions and their risk factors [34]. The information obtained from health-related physical fitness testing in PUMPING ARTERIES will:

- Educate participants about their present health/fitness status relative to health-related standards and age and sex-matched norms.
- Provide data that are helpful in development of individualized exercise prescriptions to address all health/fitness components.
- Motivating participants to establish reasonable and attainable health/fitness goals

In addition, increasing evidence points out at vascular stiffness (and in particular aortic stiffness measured by pulse wave velocity) as a reliable biomarker of vascular aging, able to integrate in a single measure the overall burden of cardiovascular risk factors on the vasculature over time [56]. Increased aortic stiffness has been shown to predict future cardiovascular events and improve risk reclassification in those at intermediate risk. In PUMPING ARTERIES, participants will have the opportunity to learn basic aspects of physiology of large artery stiffness and will be given personalized feedback on the clinical significance of the measured values compared to standards and age and sex-matched norms.

Training of specialists in the areas of research, technology and health services

Undergraduate health-science education has been undergoing a much-needed transformation to better prepare learners to provide care in complex health systems and to address anticipated workforce needs. Curricular structure and pedagogy have also been evolving with more schools challenging classic structures [67]. What is currently needed of undergraduate health-science education is to create a 21st century multidisciplinary workforce that is able to improve the health of patients and communities. Therefore, Health Science schools should additionally refocus research priorities on areas that will alter public health and patient outcomes, rather than focusing primarily on areas that have little if any utility for the majority of

the population. Thus, PUMPING ARTERIES represents a balance between workforce preparation and research suited to the needs of our communities. PUMPING ARTERIES will allow train and constitute a valuable workforce of specialized technicians, teaching them the right kind of content and competencies, using the best techniques.

Fostering harmonization between university and business

A general and uncontested view is that the research at PUMPING ARTERIES is also motivated by its relevance for education and teaching. Research should contribute to the actuality and the quality of education as well as the anchoring of education in professional practice.

Students may gain experience of applied research and experimental development through which they may enhance their competencies in the world of work. There is some evidence to support the view that students who participated in research projects or were exposed to enquiry-based learning enhanced their competencies regarding problem statement, analytical thinking, and taking a more independent perspective – all qualifications highly rewarded by employers [39]. But for those already in working life the research function of PUMPING ARTERIES is also an important dimension in enhancing key competencies. Universities may employ part-time master students in PUMPING ARTERIES for example. These students carry out real research work applied in a business setting. This enriches their education by providing additional practical and research experience. This is beneficial for PUMPING ARTERIES, GCP or any other employment organization concerned.

Privacy and Confidentiality

In order to assure the confidentiality of the participants, an identification code will be attributed to each participant in the database and all the equipment's and sheets used.

Each participant or its tutor will sign an informed consent authorizing the use of the provided contact details on a follow-up contact to schedule a new measurement round.

Storage

The database will be stored in the GCP private server.

Patient's individual reports will be completed after last assessments, including, but not limited to, quantitative and qualitative information on the following tests: aerobic fitness, body composition, arterial stiffness, resting and activity energy expenditure.

Statistical Analysis and Data Monitorization

Based upon a medium effect size of 0.154 derived from published changes in aortic PWV within-between modes of exercise [18], an a priori power analysis suggested that 22 participants are required to detect significant differences within-between groups, conditions and time points ($1\text{-}\beta = 80\%$, $\alpha = 0.05$). All data will be collated and/or analyzed using Microsoft Excel (v15.29.1, Microsoft Corporation, Redmond, Washington, United States) and Statistical Package for the Social Sciences version 24 (IBM, Armonk, New York, United States) software with results presented as mean ± SD. Due to technical issues, missing data (~4–5%) may be evident during the study and subsequently should be replaced by averaging values before and after the missing value for the relevant variable prior to analysis.

Comparisons between interventions (Bike, Pump Power, Global Training and Control) over time (Rest vs. post-intervention time points) for indices of autonomic and vascular function will be examined via two-way

(intervention × time), repeated measures analysis of variance (ANOVA) and adjusted for sex and activity energy expenditure. Session differences for indices of autonomic and vascular function and energy expenditure will be analyzed using a one-way repeated measures ANOVA. Post-hoc comparisons will be conducted via Bonferroni's tests. Statistical significance will be set at $p < 0.05$.

The database will be stored in the GCP private server throughout the study period. The GCP lab will be responsible for management, storage and is the single user of the complete database.

Compensation and Insurance

There is no compensation for any kind of expense that might be associated with the participation in PUMPING ARTERIES. However, participants are covered by the GCP's insurance plan in case of a negative event that might be associated with the participation in PUMPING ARTERIES, regardless of the affiliation status to the GCP at the time of the new measurement round.

End-of-Study Procedures

The huge database will be primarily used to answer relevant research questions regarding the longitudinal relationship between lifestyle and cardiovascular health. These results will be published in the proceedings of international scientific meetings and scientific journals within the Q1 and Q2 of Sports Science, Cardiac & Cardiovascular systems, and Peripheral Vascular Disease.

Participants of this study will be informed of the scientific publications that resulted from this project by timed publications at the GCP website (www.gcp.pt).

Conflict of Interest

The researchers have no competing interests.

References

1. Naka, K.K., et al., *Arterial distensibility: acute changes following dynamic exercise in normal subjects.* Am J Physiol Heart Circ Physiol, 2003. **284**(3): p. H970-8.
2. Hamilton, P.K., et al., *Arterial stiffness: clinical relevance, measurement and treatment.* Clin Sci (Lond), 2007. **113**(4): p. 157-70.
3. Girerd, X., et al., *Arterial distensibility and left ventricular hypertrophy in patients with sustained essential hypertension.* Am Heart J, 1991. **122**(4 Pt 2): p. 1210-4.
4. Blacher, J., et al., *Impact of aortic stiffness on survival in end-stage renal disease.* Circulation, 1999. **99**(18): p. 2434-9.
5. McEniery, C.M., *Novel therapeutic strategies for reducing arterial stiffness.* Br J Pharmacol, 2006. **148**(7): p. 881-3.
6. Vlachopoulos, C., K. Aznaouridis, and C. Stefanadis, *Prediction of cardiovascular events and all-cause mortality with arterial stiffness: a systematic review and meta-analysis.* J Am Coll Cardiol, 2010. **55**(13): p. 1318-27.
7. Charkoudian, N. and J.A. Rabbitts, *Sympathetic neural mechanisms in human cardiovascular health and disease.* Mayo Clin Proc, 2009. **84**(9): p. 822-30.
8. Fei, D.Y., et al., *Relationship between arterial stiffness and heart rate recovery in apparently healthy adults.* Vasc Health Risk Manag, 2005. **1**(1): p. 85-9.
9. van Ittersum, F.J., et al., *Autonomic nervous function, arterial stiffness and blood pressure in patients with Type I diabetes mellitus and normal urinary albumin excretion.* J Hum Hypertens, 2004. **18**(11): p. 761-8.
10. Buchheit, M. and C. Gindre, *Cardiac parasympathetic regulation: respective associations with cardiorespiratory fitness and training load.* Am J Physiol Heart Circ Physiol, 2006. **291**(1): p. H451-8.
11. Cole, C.R., et al., *Heart-rate recovery immediately after exercise as a predictor of mortality.* N Engl J Med, 1999. **341**(18): p. 1351-7.
12. Kannankeril, P.J., et al., *Parasympathetic effects on heart rate recovery after exercise.* J Investig Med, 2004. **52**(6): p. 394-401.
13. Tsuji, H., et al., *Impact of reduced heart rate variability on risk for cardiac events. The Framingham Heart Study.* Circulation, 1996. **94**(11): p. 2850-5.
14. Mahmud, A. and J. Feely, *Beta-blockers reduce aortic stiffness in hypertension but nebivolol, not atenolol, reduces wave reflection.* Am J Hypertens, 2008. **21**(6): p. 663-7.
15. Aronson, D. and A.J. Burger, *Effect of beta-blockade on heart rate variability in decompensated heart failure.* Int J Cardiol, 2001. **79**(1): p. 31-9.
16. Harvey, R.E., et al., *Influence of sympathetic nerve activity on aortic hemodynamics and pulse wave velocity in women.* Am J Physiol Heart Circ Physiol, 2017. **312**(2): p. H340-H346.
17. Kingwell, B.A., et al., *Arterial compliance increases after moderate-intensity cycling.* Am J Physiol, 1997. **273**(5 Pt 2): p. H2186-91.
18. Heffernan, K.S., et al., *Arterial stiffness and baroreflex sensitivity following bouts of aerobic and resistance exercise.* Int J Sports Med, 2007. **28**(3): p. 197-203.
19. Munir, S., et al., *Exercise reduces arterial pressure augmentation through vasodilation of muscular arteries in humans.* Am J Physiol Heart Circ Physiol, 2008. **294**(4): p. H1645-50.
20. Millen, A.M., A.J. Woodiwiss, and G.R. Norton, *Post-exercise effects on aortic wave reflection derived from wave separation analysis in young- to middle-aged pre-hypertensives and hypertensives.* Eur J Appl Physiol, 2016. **116**(7): p. 1321-9.
21. Teixeira, L., et al., *Post-concurrent exercise hemodynamics and cardiac autonomic modulation.* Eur J Appl Physiol, 2011. **111**(9): p. 2069-78.
22. Kliszczewicz, B.M., et al., *Autonomic Responses to an Acute Bout of High-Intensity Body Weight Resistance Exercise vs. Treadmill Running.* J Strength Cond Res, 2016. **30**(4): p. 1050-8.

23. de Paula, T., et al., *Acute Effect of Aerobic and Strength Exercise on Heart Rate Variability and Baroreflex Sensitivity in Men with Autonomic Dysfunction.* J Strength Cond Res, 2017.
24. Luttrell, M.J. and J.R. Halliwill, *Recovery from exercise: vulnerable state, window of opportunity, or crystal ball?* Front Physiol, 2015. **6**: p. 204.
25. da Nobrega, A.C., *The subacute effects of exercise: concept, characteristics, and clinical implications.* Exerc Sport Sci Rev, 2005. **33**(2): p. 84-7.
26. Pierce, D.R., et al., *Influence of Exercise Mode on Post-exercise Arterial Stiffness and Pressure Wave Measures in Healthy Adult Males.* Front Physiol, 2018. **9**: p. 1468.
27. Michael, S., K.S. Graham, and G.M. Davis, *Cardiac Autonomic Responses during Exercise and Post-exercise Recovery Using Heart Rate Variability and Systolic Time Intervals-A Review.* Front Physiol, 2017. **8**: p. 301.
28. Cunha, F.A., et al., *Parasympathetic reactivation after maximal CPET depends on exercise modality and resting vagal activity in healthy men.* Springerplus, 2015. **4**: p. 100.
29. Maeder, M.T., et al., *Impact of the exercise mode on heart rate recovery after maximal exercise.* Eur J Appl Physiol, 2009. **105**(2): p. 247-55.
30. Rahimi, K., et al., *Implications of exercise test modality on modern prognostic markers in patients with known or suspected coronary artery disease: treadmill versus bicycle.* Eur J Cardiovasc Prev Rehabil, 2006. **13**(1): p. 45-50.
31. Abrantes, C., et al., *Physiological responses to treadmill and cycle exercise.* Int J Sports Med, 2012. **33**(1): p. 26-30.
32. Hill, D.W., J.N. Halcomb, and E.C. Stevens, *Oxygen uptake kinetics during severe intensity running and cycling.* Eur J Appl Physiol, 2003. **89**(6): p. 612-8.
33. Jamison, J.P., J. Megarry, and M. Riley, *Exponential protocols for cardiopulmonary exercise testing on treadmill and cycle ergometer.* Eur J Appl Physiol, 2010. **108**(1): p. 167-75.
34. Riebe, D., et al., *ACSM's guidelines for exercise testing and prescription*. 10th ed. 2018, Philadelphia.
35. Dasilva, S.G., et al., *Psychophysiological responses to self-paced treadmill and overground exercise.* Med Sci Sports Exerc, 2011. **43**(6): p. 1114-24.
36. B.C., F. *Affective responses to 10-minute and 30-minute walks in sedentary, overweight women: Relationships with theory-based correlates of walking for exercise*. Psychol, 2013. **14**, 759–766.
37. Augustine, J., et al., *Effect of a single bout of resistance exercise on arterial stiffness following a high-fat meal.* Int J Sports Med, 2014. **35**(11): p. 894-9.
38. Compher, C., et al., *Best practice methods to apply to measurement of resting metabolic rate in adults: a systematic review.* J Am Diet Assoc, 2006. **106**(6): p. 881-903.
39. Garber, C.E., et al., *American College of Sports Medicine position stand. Quantity and quality of exercise for developing and maintaining cardiorespiratory, musculoskeletal, and neuromotor fitness in apparently healthy adults: guidance for prescribing exercise.* Med Sci Sports Exerc, 2011. **43**(7): p. 1334-59.
40. Battista, R.A., et al., *Physiologic responses during indoor cycling.* J Strength Cond Res, 2008. **22**(4): p. 1236-41.
41. Pouliot, M.C., et al., *Waist circumference and abdominal sagittal diameter: best simple anthropometric indexes of abdominal visceral adipose tissue accumulation and related cardiovascular risk in men and women.* Am J Cardiol, 1994. **73**(7): p. 460-8.
42. McLaughlin, J., et al., *Validation of the COSMED K4 b2 portable metabolic system.* Int J Sports Med, 2001. **22**(4): p. 280-4.
43. Bogert, L.W., et al., *Reconstruction of brachial pressure from finger arterial pressure during orthostasis.* J Hypertens, 2004. **22**(10): p. 1873-80.
44. Cymberknop, L.J., et al., *Modeling young and adult patients with cirrhosis through a three element windkessel (WK3e).* Conf Proc IEEE Eng Med Biol Soc, 2017. **2017**: p. 266-269.
45. Sagawa, K., et al., *Cardiac Contraction and the Pressure-volume Relationship.* 1 ed. 1988, New

York: Oxford University Press

46. Segers, P., et al., *Three- and four-element Windkessel models: assessment of their fitting performance in a large cohort of healthy middle-aged individuals.* Proc Inst Mech Eng H, 2008. **222**(4): p. 417-28.
47. Mendonca, G.V., et al., *Sex differences in linear and nonlinear heart rate variability during early recovery from supramaximal exercise.* Appl Physiol Nutr Metab, 2010. **35**(4): p. 439-46.
48. Pagani, M., et al., *Power spectral analysis of heart rate and arterial pressure variabilities as a marker of sympatho-vagal interaction in man and conscious dog.* Circ Res, 1986. **59**(2): p. 178-93.
49. *Heart rate variability: standards of measurement, physiological interpretation and clinical use. Task Force of the European Society of Cardiology and the North American Society of Pacing and Electrophysiology.* Circulation, 1996. **93**(5): p. 1043-65.
50. Westerhof, B.E., et al., *Time-domain cross-correlation baroreflex sensitivity: performance on the EUROBAVAR data set.* J Hypertens, 2004. **22**(7): p. 1371-80.
51. O'Rourke, M.F., et al., *Clinical applications of arterial stiffness; definitions and reference values.* Am J Hypertens, 2002. **15**(5): p. 426-44.
52. Liang, Y.L., et al., *Non-invasive measurements of arterial structure and function: repeatability, interrelationships and trial sample size.* Clin Sci (Lond), 1998. **95**(6): p. 669-79.
53. Sugawara, M., et al., *Relationship between the pressure and diameter of the carotid artery in humans.* Heart Vessels, 2000. **15**(1): p. 49-51.
54. Swampillai, J., et al., *Acute effects of caffeine and tobacco on arterial function and wave travel.* Eur J Clin Invest, 2006. **36**(12): p. 844-9.
55. Rakebrandt, F., et al., *Arterial wave intensity and ventricular-arterial coupling by vascular ultrasound: rationale and methods for the automated analysis of forwards and backwards running waves.* Ultrasound Med Biol, 2009. **35**(2): p. 266-77.
56. Boutouyrie, P. and R.M. Bruno, *The Clinical Significance and Application of Vascular Stiffness Measurements.* Am J Hypertens, 2019. **32**(1): p. 4-11.
